# Coccolithophores and diatoms resilient to ocean alkalinity enhancement: A glimpse of hope?

**DOI:** 10.1126/sciadv.adg6066

**Published:** 2023-06-14

**Authors:** James A. Gately, Sylvia M. Kim, Benjamin Jin, Mark A. Brzezinski, Maria D. Iglesias-Rodriguez

**Affiliations:** ^1^ Department of Ecology, Evolution, and Marine Biology, University of California, Santa Barbara; Santa Barbara, CA 93106, USA.; ^2^ Marine Science Institute, University of California, Santa Barbara, CA 93106, USA.; ^3^ Department of Molecular, Cellular, and Developmental Biology, University of California, Santa Barbara; Santa Barbara, CA 93106, USA.

## Abstract

It is increasingly apparent that adequately mitigating anthropogenic climate interference will require ocean carbon dioxide removal (CDR) strategies. Ocean alkalinity enhancement (OAE) is an abiotic ocean CDR approach that aims to increase the ocean’s CO_2_ uptake capacity through the dispersal of pulverized mineral or dissolved alkali into the surface ocean. However, OAE’s effect on marine biota is largely unexplored. Here, we investigate the impacts of moderate (~700 μmol kg^−1^) and high (~2700 μmol kg^−1^) limestone-inspired alkalinity additions on two biogeochemically and ecologically important phytoplankton functional group representatives: *Emiliania huxleyi* (calcium carbonate producer) and *Chaetoceros* sp. (silica producer). The growth rate and elemental ratios of both taxa showed a neutral response to limestone-inspired alkalinization. While our results are encouraging, we also observed abiotic mineral precipitation, which removed nutrients and alkalinity from solution. Our findings offer an evaluation of biogeochemical and physiological responses to OAE and provide evidence supporting the need for continued research into how OAE strategies affect marine ecosystems.

## Teaser

Coccolithophores and diatoms are resilient to alkalinization, but precipitation at high alkalinities may reduce CDR’s efficacy.

## INTRODUCTION

The 2015 Paris Agreement—based on a range of scenarios assessed in the International Panel on Climate Change’s Fifth Assessment Report (*1*)—set a goal to limit “…the increase in average global temperature to well below 2°C above pre-industrial levels and to pursue efforts to limit the temperature increase to 1.5°C above pre-industrial levels” (*2*, *3*). It has been suggested that meeting this target with emission reductions and land-based carbon dioxide removal (CDR) approaches alone may not be possible and that ocean CDR approaches may be needed to achieve the required removal of ~9 Gt CO_2_/year (*4*, *5*).

Ocean alkalinity enhancement (OAE)—also referred to as artificial ocean alkalinization and enhanced/accelerated weathering—is an abiotic ocean CDR technology that has received increasing attention because of its large carbon storage potential and possible ecological benefits via mitigation of ocean acidification (OA) (*5*–*7*). This ocean CDR approach is inspired in the restoration of alkalinity through rock weathering, an Earth process that occurs naturally on geological time scales (*8*). The rationale for OAE is that the increase in total alkalinity (TA) can result in a permanent removal of CO_2_. It has been proposed that OAE can be achieved through regional dispersion of pulverized or predissolved carbonate or silicate rocks—e.g., limestone and its derivatives (CaO or Ca[OH]_2_), olivine (*5*, *9*)—or electrochemically generated forms of alkalinity into the surface ocean or along continental shelves, each of which have unique sets of chemical, logistical, and technoeconomic challenges and concerns (*5*, *6*). The increase in TA via the addition of conservative ions—i.e., ions that obey a linear mixing relationship (*10*)—locks CO_2_ in the form of bicarbonate (${\mathrm{HCO}}_{3}^{-}$) and carbonate (${\mathrm{CO}}_{3}^{2-}$) ions. OAE has the ancillary benefit of reversing OA, especially at the site of deployment before equilibration with atmospheric CO_2_, as the dissolution reactions consume protons. Thus, OAE may additionally provide a quasi-natural means of ecosystem restoration for fragile habitats affected by OA—e.g., coral reefs (*11*).

While technically feasible, the potential biological effects of OAE remain largely unexplored (*12*, *13*). Studies examining the effects of seawater alkalinization on marine biota are sparse (*11*, *14*–*17*), and—while a recent study assessed OAE’s effect on phytoplankton community succession (*17*)—presently, no empirical study has assessed its biogeochemical and physiological impacts on individual phytoplankton functional groups (PFGs). PFGs are the foundation of ocean food webs, and their unique biogeochemical properties govern the cycling of elements between the atmosphere, the upper ocean, and the ocean interior. For example, coccolithophores are predominant contributors to the production of biogenic calcite (CaCO_3_) (*18*), and thus their biogeochemical response to OAE is of particular concern as an increase in cellular calcification would reduce seawater alkalinity and release CO_2_—diminishing the efficacy of CDR; meanwhile, diatoms control the cycling of biogenic silica (BSi) in the ocean and are prominent primary producers and exporters of particulate organic carbon (POC) in marine ecosystems (*19*, *20*).

Coccolithophores and diatoms have often been studied alongside each other as they have distinct onsets in the seasonal phytoplankton succession and their unique physiological adaptations and ecological niches are apparent on both ecological and geological time scales (*21*–*23*). One recent hypothesis suggests that OAE could have a differential impact on diatoms and coccolithophores (*6*); specifically, OAE achieved through the dissolution of carbonate minerals (or their derivatives), yielding increases in [Ca^2+^] and TA, is expected to promote the growth of calcifiers while the dissolution of Si-based minerals is anticipated to benefit diatoms. A recent study found that increasing TA (without calcium addition) decreased the contribution of microphytoplankton (e.g., diatoms) to total phytoplankton biomass, as well as the production of biogenic silica by diatoms, while the proportion of nanophytoplankton (e.g., coccolithophores) increased (*17*). However, the authors noted that the complexity of ocean food webs and absence of information on species-specific abundances made interpretation of their results difficult, further emphasizing the need for species-targeted studies (*17*).

We assess the biogeochemical and physiological response to limestone-inspired OAE (i.e., Na_2_CO_3_ + CaCl_2_H_4_O_2_) in representative species of two biogeochemically important PFGs: the coccolithophore *Emiliania huxleyi*—a ubiquitous calcifier in the modern ocean that is responsible for a large proportion of CaCO_3_ production in the pelagic zone (*24*)—and the diatom *Chaetoceros* sp.—a representative of arguably the most globally abundant and diverse genus of diatoms in the modern ocean (*25*). Furthermore, we provide estimates of the ocean’s elevated CO_2_-drawdown potential under model-predicted OAE scenarios.

## RESULTS

### Effect of alkalinization on the carbonate system

Initial alkalinity spikes during an OAE experiment absent of biology reduced the partial pressure of CO_2_ (μatm) by ~50 and ~80%, increased pH by approximately 0.33 and 0.76 logarithmic units, and increased total dissolved inorganic carbon (TCO_2_) by ~20 and ~70% in moderate- and high-TA media, respectively (data S1). The large increases in [TCO_2_] observed within treatment media at the beginning of the experiment, despite the large reductions in [CO_2_], were likely driven by changes in $\left[{\mathrm{CO}}_{3}^{2-}\right]$—which were approximately 136 and 606% higher in moderate- and high-TA media than in controls, compared to a relatively smaller increase of ~10 and ~23% in $\left[{\mathrm{HCO}}_{3}^{-}\right]$, respectively (data S1). pCO_2_ in TA treatment media equilibrated with the air phase following 7 to 11 days of gentle bubbling with air containing 420 ppm (± 2%) CO_2_, reducing $\left[{\mathrm{CO}}_{3}^{2-}\right]$ and increasing $\left[{\mathrm{HCO}}_{3}^{-}\right]$ in the process (data S1).

**Fig. 1.**
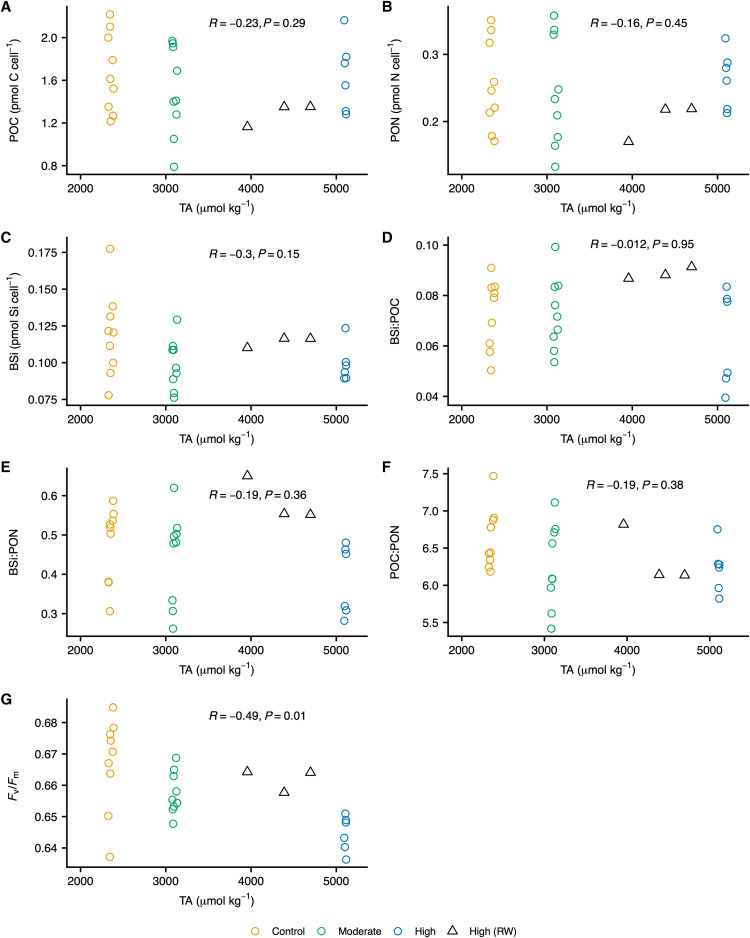
Influence of TA on *Chaetoceros* sp. biochemistry and physiology. Here, we show the effect of TA on *Chaetoceros* sp. cellular (**A**) POC (**B**) PON and (**C**) BSi; (**D**) BSi:POC, (**E**) BSi: PON, and (**F**) POC:PON elemental ratios; and (**G**) *F*_v_/*F*_m_. Each open circle represents an independent control (yellow), moderate-TA (green), or high-TA (blue) replicate. Triplicate independent replicates were collected for each treatment level on each sampling date—i.e., days 2, 3, and 4 (day 0 values were excluded due to low cell abundances). Each open black triangle represents an independent high-TA replicate that underwent reverse weathering during sample storage (these values were excluded from statistical analysis). Displayed *R* and values were calculated using Spearman’s rank-based correlation test. To avoid overplotting and ease readability, a small, random amount of variation was added to the location of each data point using the “geom_jitter ()” function in R.

Starting carbonate system conditions for each treatment level during both the *E. huxleyi* and *Chaetoceros* sp. experiments were within the range of targeted model-predicted OAE scenarios (see Materials and Methods; Table 1). Over the course of the experiments, TA trends differed between the two biotic experiments: TA decreased during the *E. huxleyi* experiment, consistent with the formation of biogenic calcite, but increased slightly during the *Chaeotoceros* sp. experiment, likely due to differential nutrient uptake—e.g., nitrate and phosphate (figs. S1A and S2A, respectively) (*10*). TA values were nearly constant during the abiotic experiment (fig. S3A). Notably, reverse weathering was observed within carbonate chemistry samples collected from high-TA treatments on day 4 of the *Chaetoceros* sp. experiment: compared with mean TA values of earlier sampling points (~5100 μmol kg^−1^ in the high-TA treatments), the mean TA for day 4 was 4350 ± 371 μmol kg^−1^—a TA decline of ~750 μmol kg^−1^ (fig. S2A). Alongside the reduction in TA, secondary precipitates—i.e., minerals white in appearance—were visible in day 4’s carbonate chemistry sample bottles. Although these values were excluded from all statistical analyses, we identify these affected data points in relevant figures (i.e., Fig. 1, A to G, and fig. S2, A to G).

**Table 1.** Carbonate chemistry parameters at the beginning of OAE culturing experiments. Mean values are given with 1 SD in parentheses (*n* = 3).

Experiment	OAE group	TA (μmol kg^−1^)	TCO_2_ (μmol kg^−1^)	pH	pCO_2_ (μatm)	${\mathrm{HCO}}_{3}^{-}$(μmol kg^−1^)	${\mathrm{CO}}_{3}^{2-}$(μmol kg^−1^)	CO_2_ (μmol kg^−1^)	Ω_ca_
*Emiliania huxleyi*	Control	2320 (6)	2120 (5)	8.01 (0.002)	455 (2)	1960 (4)	143 (0.7)	17 (0.06)	3.44 (0.02)
	Moderate	3050 (5)	2720 (10)	8.15 (0.02)	406 (20)	2460 (20)	251 (7)	15.2 (0.6)	6.06 (0.2)
	High	4830 (400)	4050 (300)	8.42 (0.04)	303 (30)	3400 (200)	646 (90)	11.4 (1)	15.6 (2)
*Chaetoceros* sp.	Control	2330 (4)	2120 (2)	8.03 (0.005)	430 (5)	1950 (2)	150 (2)	16.1 (0.2)	3.63 (0.04)
	Moderate	3080 (2)	2730 (10)	8.17 (0.02)	391 (20)	2460 (20)	261 (8)	14.6 (0.6)	6.31 (0.2)
	High	5070 (40)	4240 (70)	8.44 (0.03)	302 (30)	3530 (90)	699 (20)	11.3 (0.9)	16.9 (0.6)

pH values trended upward during both biotic experiments, but the increase was more pronounced in the diatom cultures (figs. S1C and S2C). For example, average pH in control and moderate-TA treatments during the *Chaetoceros* sp. experiment increased by ~0.49 and ~0.27 from day 0 to day 4 while increasing by only ~0.15 and ~0.06 by day 6 during the *E. huxleyi* experiment, respectively; this difference can be attributed to the more pronounced decrease in [pCO_2_] in *Chaetoceros* sp. cultures relative to those of *E. huxleyi* (figs. S1D and S2D). pH decreased over the course of the abiotic experiment, consistent with CO_2_ ingassing (fig. S3C). Although pCO_2_ concentrations largely declined over the course of the biotic experiments—with the exception of high-TA treatments between days 0 and 5 of the *E. huxleyi* experiment and days 0 and 2 of the *Chaetoceros* sp. experiment, which showed increases in [pCO_2_]—the decline was more pronounced in the controls, likely a result of CO_2_ ingassing from the 420 ppm CO_2_ supplied air initially outpacing photosynthesis as [pCO_2_] increased rapidly in both TA treatments during the abiotic experiment (figs. S1D, S2D, and S3D).

The concentration of ${\mathrm{HCO}}_{3}^{-}$ trended downward during both the *E. huxleyi* and *Chaetoceros* sp. experiment (figs. S1E and S2E)—likely due to the photosynthetic uptake of TCO_2_ by both organisms and, in the case of *E. huxleyi*, calcification—but we observed differential responses between the two experiments for both the concentration of ${\mathrm{CO}}_{3}^{2-}$ and, by association, calcite saturation state (Ω_ca_). Both parameters remained relatively unaltered during the *E. huxleyi* experiment (fig. S1, F and G). However, during the *Chaetoceros* sp. experiment, while $\left[{\mathrm{CO}}_{3}^{2-}\right]$ and Ω_ca_ increased in the control and moderate-TA cultures, both declined in the high-TA treatments through day 2, after which both began trending upward until reverse weathering affected samples collected on the final day of the experiment (fig. S2, F and G). $\left[{\mathrm{HCO}}_{3}^{-}\right]$, [${\mathrm{CO}}_{3}^{2-}]$, and Ω_ca_ remained relatively unaltered in control and moderate-TA treatments during the abiotic experiment; however, in the high-TA treatment, until [CO_2_] in the media approached equilibrium with the air phase, [${\mathrm{HCO}}_{3}^{-}]$ increased and [${\mathrm{CO}}_{3}^{2-}]$ and Ω_ca_ decreased (fig. S3, E to G)—consistent with acidification from ingassing.

### Trends in nutrient evolution

**Fig. 2.**
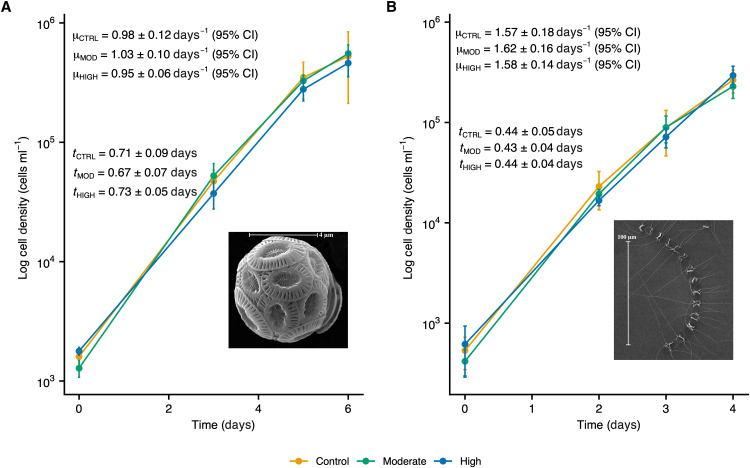
Growth rates of *Emiliania huxleyi* and *Chaetoceros* sp. The graphs display *Emiliania huxleyi* (**A**) and *Chaetoceros* sp. (**B**) log cell abundances (cells per milliliter) for our control (yellow), moderate-TA (green), and high-TA (blue) cultures throughout each experimental timeframe. Specific growth rates (μ), generation times (*t*), and scanning electron microscopy images are displayed within plot axes. Error bars represent one SD (*n* = 3). CI, confidence interval.

Our control and treatment cultures remained largely replete with nutrients for the duration of both the *E. huxleyi* and *Chaetoceros* sp. culturing experiments—although we likely observed the onset of nutrient depletion for dissolved inorganic phosphate (DIP) on the last day of the *Chaetoceros* sp. experiment (table S1). However, the effects of potential DIP limitation were not reflected in *Chaetoceros* sp. growth rates or cellular physiology (Figs. 2B and 1G). Notably, we observed measurable differences in the concentrations of both DIP and dissolved silicic acid (DSi, Si[OH]_4_) at the beginning (day 0) of both culturing experiments. The concentration of DIP in the moderate-TA cultures was similar to that observed in the control during the *E. huxleyi* experiment, but 4% higher in the high-TA cultures (Table 2). Initial DIP concentrations were 1 and 8% higher in the moderate- and high-TA treatments at the beginning of the *Chaetoceros* sp. experiment, respectively (Table 2). DSi concentrations were between 5 and 18% lower in the treatment cultures at the beginning of both experiments (Table 2). Although DSi concentrations were typically lower in *Chaetoceros* sp. cultures with high TA values throughout the experiment, concentrations remained above what would be considered limiting to most diatoms (table S1) (*26*).

**Table 2.** Differences in DIP and DSi concentrations on day 0 of OAE culturing experiments. Mean values are given with 1 SD in parentheses (*n* = 3). Differences are relative to controls.

Experiment	OAE group	DIP (μM)	ΔDIP (%)	DSi (μM)	ΔDSi (%)
*Emiliania huxleyi*	Control	4.69 (0.06)	–	71.63 (1.03)	–
	Moderate	4.69 (0.02)	0 (NaN)	68.3 (0.2)	−5 (1)
	High	4.9 (0.03)	4 (2)	59.1 (0.17)	−18 (1)
*Chaetoceros* sp.	Control	4.49 (0.05)	–	71.37 (0.35)	–
	Moderate	4.55 (0.03)	1 (2)	66.65 (0.12)	−7 (1)
	High	4.85 (0.05)	8 (2)	59.27 (0.26)	−17 (2)

**Fig. 3.**
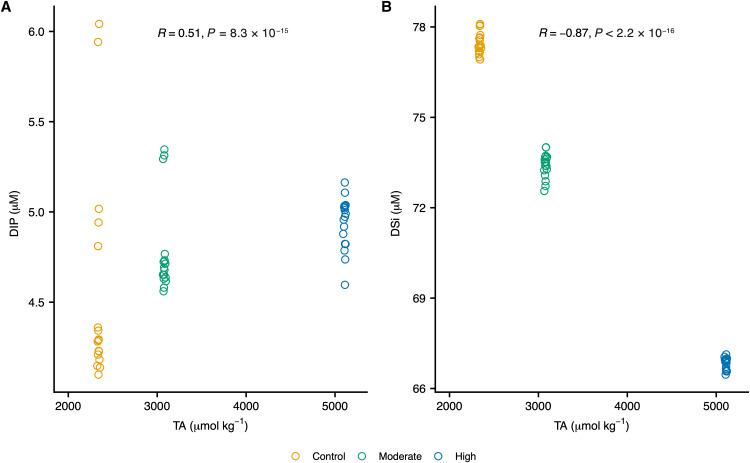
Influence of TA on nutrient concentrations. The graphs show concentrations of (**A**) DIP and (**B**) DSi during our abiotic ocean alkalinity enhancement experiment. Each open circle represents an independent control (yellow), moderate-TA (green), or high-TA (blue) replicate. Triplicate independent replicates were collected for each treatment level on each sampling date—i.e., days 0, 3, 5, 6, 7, and 11 (note: one control replicate was lost on day 0). Data points in this figure were jittered to avoid overplotting. Displayed R and *P* values were calculated using Spearman’s rank-based correlation test. To avoid overplotting and ease readability, a small, random amount of variation was added to the location of each data point using the “geom_jitter ()” function in R.

While the influence of biology and its reciprocal interactions with seawater chemistry prevented us from making conclusions about nutrient trends as a function of TA during our biotic experiments, the abiotic experiment revealed statistically significant correlations between dissolved inorganic nutrients and TA. We observed a moderately positive correlation between TA and DIP (Fig. 3A) and a strongly negative correlation with DSi (Fig. 3B). Average DIP concentrations in the moderate- and high-TA treatments were up to 11 and 19% higher relative to the control, respectively (table S2). In contrast to DIP, average DSi concentrations were 5 and 14% lower than the control in the moderate- and high-TA treatments, respectively (table S2).

### Physiological and biogeochemical responses of *E. huxleyi* and *Chaetoceros* sp.

**Fig. 4.**
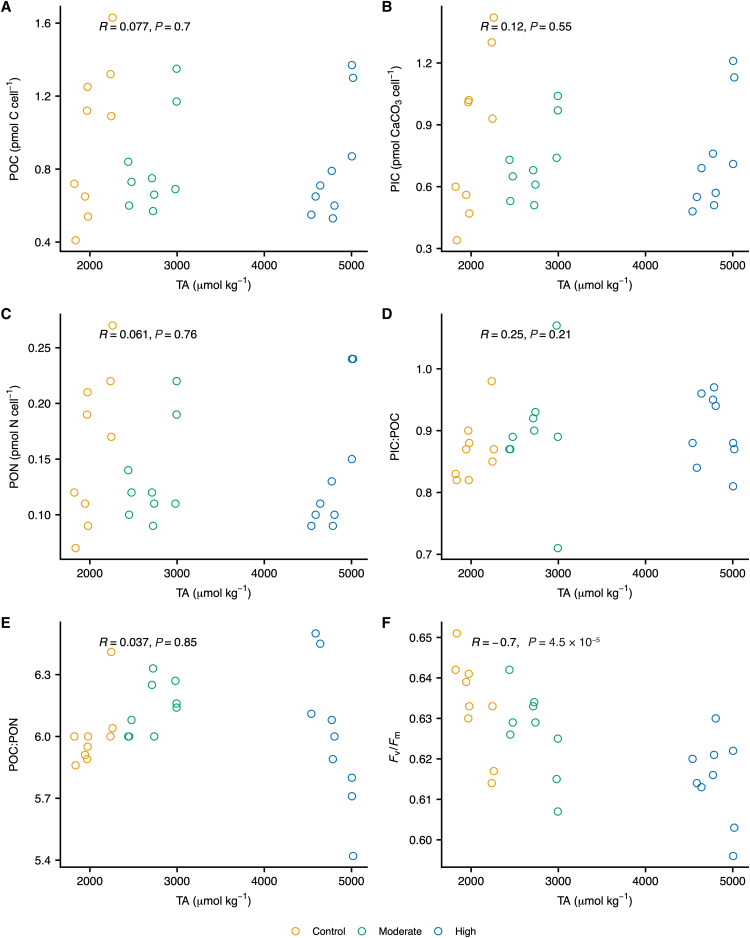
Influence of TA on *Emiliania huxleyi* biochemistry and physiology. Here, we show the effect of TA on *E. huxleyi* cellular (**A**) POC, (**B**) PIC, and (**C**) PON; (**D**) PIC:POC and (**E**) POC:PON elemental ratios; and (**F**) *F*_v_/*F*_m_. Each open circle represents an independent control (yellow), moderate-TA (green), or high-TA (blue) replicate. Triplicate independent replicates were collected for each treatment level on each sampling date—i.e., days 3, 5, and 6 (day 0 values were excluded due to low cell abundances). Displayed *R* and *P* values were calculated using Spearman’s rank-based correlation test.

The specific growth rates (μ, days^−1^) of *E. huxleyi* (Fig. 2A) and *Chaetoceros* sp. (Fig. 2B) were well constrained and comparable to those of the control in both the moderate- and high-TA treatments. No statistically significant correlation was observed between *E. huxleyi* cellular particulate inorganic carbon (PIC), cellular POC, particulate organic nitrogen (PON), PIC:POC, or POC:PON and TA (Fig. 4, A to E). However, standing stock PIC and POC values showed high variability among replicates. We likewise found no significant differences in *Chaetoceros* sp. cellular POC, PON, or BSi content as function of TA (Fig. 1, A to F). Notably, we observed a moderately negative, statistically significant correlation between *F*_v_/*F*_m_ and TA in both species (Figs. 4F and 1G). In the high-TA treatments, mean *F*_v_/*F*_m_ for *E. huxleyi* and *Chaetoceros* sp. were 2 and 3% lower than those in the controls, respectively (table S3). It should be noted, however, that *F*_v_/*F*_m_ values remained within the range typically observed for nutrient-replete, healthy phytoplankton cells in all cultures (*27*).

## DISCUSSION

Extensive experimentation on the biological responses to changes in seawater carbonate chemistry and pH have focused on OA (*28*–*30*), while the biogeochemical responses to ocean alkalinization are largely unknown. It is reasonably well established that adding alkalinity to the surface ocean has the potential to lock CO_2_ into other forms of dissolved inorganic carbon, and therefore promote an influx of atmospheric CO_2_ into the ocean, but the effects of OAE on biota remain an open question (*5*). Our study addresses a key research and development need (*5*) in ecologically and geologically important biomineral-producing PFGs that have a prominent role in the global cycling of carbon.

We report that limestone-inspired alkalinization—which increased TA concentrations well above reported surface ocean variations of approximately 2150 to 2450 μmol kg^−1^ (*31*)—had undetectable effects on the biogeochemical properties and modest effects (≤ 3% difference compared to the controls) on the photosynthetic quantum yield of two key PFGs: coccolithophores and diatoms. Our findings suggest that calcifiers and silicifiers may be resilient to seawater alkalinization, at least when pH fluctuations are within the natural variability of typical coastal and open ocean waters (*32*, *33*).

Our findings stand in contrast with the idea that the dissolution of calcium-containing alkali would benefit calcifiers (*6*). Instead, our observations using a bloom-forming, ecologically relevant coccolithophore species revealed no measurable biogeochemical differences under increasing [Ca^2+^] and TA, and modest decreases in photosynthetic quantum yield. As natural analogs of ecosystems with lower (*34*) and higher (*35*) TAs relative to average ocean TAs, respectively, the Baltic and Black Seas have been the focus of study of the potential ecological effects of OAE in the context of coccolithophore dynamics because, in these brackish environments, the coccolithophore *E. huxleyi* is abundant in the Black Sea but absent from the Baltic Sea (*36*). A shift in the Black Sea’s phytoplankton community makeup has also been reported, with coccolithophores increasing in abundance in a system previously dominated by diatoms and flagellates (*37*, *38*). On the basis of our observations, and previous work indicating the complexity of factors governing the prominence of PFGs (*21*, *22*, *39*), factors other than TA—e.g., variations in nutrient fluxes, including lower silicate loading into the Black Sea following the damming of the River Danube (*37*, *40*)—are likely at least partly responsible for the reported shifts in the Black Sea’s phytoplankton community composition (*37*, *38*).

The lack of measurable differences in PIC:POC (Fig. 4D)—the ratio of calcification to organic carbon fixation, which is used to characterize calcification as either a sink (conservatively when PIC:POC < 1) or source (conservatively when PIC:POC > 1) of CO_2_ (*41*)—indicates that *E. huxleyi* remained a sink for CO_2_ under the TA values used in our experiments. It is possible, however, that the response of coccolithophores to seawater alkalinization might be species-specific. It appears that the gradual decline in atmospheric CO_2_ concentrations following the Paleocene–Eocene Thermal Maximum, which was likely characterized by a steady increase in seawater alkalinity partly due to mineral weathering (*8*), resulted in divergent responses in coccolithophorid calcification–to–carbon fixation ratios as a function of cell size (*42*). Although we are presently unable to provide an explanation for the high variability that we observed in *E. huxleyi* standing stocks of cellular PIC and POC, similar variability has been reported in *E. huxleyi* in response to changes in [CO_2_] (*43*) and temperature (*44*). We also point out that the number of independent replicates collected during each of our experiments is adequate for revealing trends as a function of TA, but none were observed for either PIC or POC.

The lack of measurable differences in the production of BSi in our *Chaetoceros* sp. cultures suggests that diatom silicification might similarly be unaffected by seawater alkalinization. Recent research reported that seawater alkalinization reduced DSi drawdown and BSi production by diatoms, but the authors note their difficulty in determining whether TA’s effect on diatom BSi content was direct or indirect (*17*). The lack of measurable differences in *Chaetoceros* sp. BSi content (Fig. 1, C to E), and the negative correlation that we observed between DSi and elevated TA (Fig. 3B), indicates that TA’s effect on diatom silica content might be indirect—e.g., if seawater alkalinization reduces DSi concentrations below threshold concentrations, it is possible that diatom silicification rates and community composition could be affected; alternatively, if silicate rocks are used to achieve OAE, the resulting increase in DSi might favor diatom growth. It is also important to note that the response of diatoms to OAE might vary among species. For example, species-specific responses in diatoms have been reported due to OA (*45*, *46*).

Although our findings demonstrate that limestone-inspired alkalinity enhancement has little effect on coccolithophore and diatom physiology and biogeochemistry, one must note that these experiments only considered the short-term acclimation of *E. huxleyi* and *Chaetoceros* sp. and that selection might occur on longer time scales. Notably, within these short timeframes, we observed a negative correlation between photosynthetic quantum yield and TA in both species; it is possible, then, that an increase in TA—and the accompanying shifts in carbonate chemistry—might yield a modest reduction in photosynthetic quantum yield. However, the impact of a decreased *F*_v_/*F*_m_ was not manifested in the cellular production of POC (Figs. 4A and 1A) or in growth rates (Fig. 2).

One may note that the *F*_v_/*F*_m_ values for *Chaetoceros* sp. in the reverse-weathering samples shown in Fig. 1G are higher than those of other high-TA replicates, which suggests that *Chaetoceros* sp. cells were healthier after reverse weathering. However, although we cannot be certain, we suspect that reverse weathering of day 4’s carbonate chemistry samples occurred during storage and that *Chaetoceros* sp. cells were not subjected to reverse weathering while in culture (see discussion on secondary mineral precipitates below). If reverse weathering did indeed occur during sample storage and *Chaetoceros* sp. cells were not exposed to reverse weathering while in culture, the higher *F*_v_/*F*_m_ values recorded on day 4 of the experiment are within the SD of those reported in *Chaetoceros* sp. control and moderate-TA cultures (table S3).

Scanning electron microscopy with energy-dispersive x-ray (SEM-EDX) analysis of samples collected from our high-TA treatments during the abiotic OAE experiment revealed mineral precipitates that were partially composed of Fe, Si, and P (figs. S4 to S6), indicating that mineral precipitation caused by seawater alkalinization removes inorganic nutrients from solution. The variations in dissolved nutrient concentrations that we observed during our abiotic laboratory experiment lends further support to this hypothesis (Fig. 3). The opposing effects of TA on DSi relative to DIP is particularly interesting. While the mechanism(s) driving the differential response between DSi and DIP at higher TAs remains unclear, it is possible that seawater alkalinization’s effect on pH is at least partly responsible for the observed responses. Although relative differences in DSi concentrations between treatments and controls vary little following the initial alkalinity spikes, relative differences between DIP concentrations in treatments and controls vary until pH stabilizes after ~5 days of bubbling (table S2 and data S1). Recent research has suggested that phosphate absorption onto hydrous ferric oxides typically occurs more easily at lower pH values (*47*). The DIP trends we observed during the abiotic experiment—along with the presence of iron oxides in mineral precipitates analyzed via SEM-EDX (figs. S4, S5, and S7)—point to iron oxide formation as a potential explanation for DIP removal as DIP concentrations were typically higher in our high-TA (and thus higher pH) treatments relative to controls. However, initial limestone-inspired alkalinity spikes—which caused pH values to reach 8.44 and 8.87 in moderate- and high-TA media, respectively—produced the opposite trend: DIP concentrations were lower in the higher-TA media compared to controls (table S2). Ca^2+^ can reportedly contribute to P removal when pH values reach >8.5 (*47*), so it is possible that Ca-P precipitates formed after the initial alkalinity spikes, which were accompanied by the addition of 0.38 and 1.43 mM Ca^2+^ in the moderate- and high-TA treatments, respectively. Ca and P were observed in mineral precipitates found in high-TA treatment media (figs. S4 and S5). Although the effect of alkalinization on DSi and DIP concentrations that we observed during our experiments, as well as SEM-EDX analysis of mineral precipitates found in high-TA treatment media during our abiotic experiment, indicate seawater alkalinization could potentially remove inorganic nutrients from solution, nutrient concentrations in the surface ocean are markedly lower than those of our nutrient-enriched media (*31*); thus, it remains to be seen whether similar nutrient losses occur under natural conditions.

Our results revealed that seawater alkalinization can also lead to the formation of secondary precipitates (i.e., occurring after the collection of carbonate chemistry samples; see Materials and Methods), which is an undesirable side effect of OAE. Decreases in TA caused by reverse weathering of carbonate minerals following seawater alkalinization has recently been reported (*48*). Specifically, we observed white-colored mineral precipitates within carbonate chemistry samples collected from high-TA treatments on the final day of the *Chaetoceros* sp. experiment—likely a carbonate-based precipitate based on reduced concentrations of carbonate ions and a lower Ω_ca_ (fig. S2, F and G). These secondary mineral precipitates were not, however, observed within any carbonate chemistry samples collected during our *E. huxleyi* and abiotic experiments, within any control or moderate-TA samples during our *Chaetoceros* sp. experiment or within high-TA samples collected at earlier stages of *Chaetoceros* sp. growth. It is reasonable to assume, then, that perhaps the greater rise in pH and more extreme carbon chemistry conditions (lower CO_2_ and higher CO_3_^2−^ and HCO_3_^−^ concentrations) at the end of the *Chaetoceros* sp. experiment led to reverse weathering (fig. S2). While it is possible that *Chaetoceros* sp. photosynthetic activity might have contributed to the conditions leading to precipitation, we lack the evidence to say so with certainty. Notably, we did not observe any secondary mineral precipitates within day 4’s high-TA carbonate chemistry samples on the day of collection, suggesting instead that reverse weathering occurred over time in storage. We gently filtered all carbonate chemistry samples during sample collection (see Materials and Methods) to remove particles that precipitated within culture replicates (discussed in the previous paragraph) and phytoplankton cells, providing further support to this hypothesis.

Other OAE approaches must be explored to identify optimal approaches and methods and to determine the extent to which OAE as an ocean CDR strategy is an acceptable way to enhance oceanic CO_2_ uptake while promoting ecosystem restoration via mitigation of OA. Our results provide a reference for alkalinity thresholds to avoid reverse weathering—a process that has multifold impacts including reduction of CDR potential, removal of inorganic nutrients, and physical impacts on marine life—and highlights the need to more definitively establish these thresholds. In addition, our use of Na_2_CO_3_ + CaCl_2_H_4_O_2_ to reach targeted TA values—which we selected due to the slow and incomplete dissolution of proposed ocean-liming minerals (e.g., CaO) during laboratory tests—stresses the need to prioritize the development of engineering strategies to effectively alkalinize seawater. The dissolution of other proposed OAE minerals (e.g., olivine) have also displayed slow dissolution rates (*49*).

Our laboratory experiments are a step toward understanding ecosystem responses to OAE and offer a glimpse of how biogeochemically and ecologically important PFGs that are central to the cycling of carbon on planetary scales respond to limestone-inspired seawater alkalinization. Assessment of how other PFGs (e.g., nitrifiers and toxin producers), and especially phytoplankton community assemblages, respond to calcium-containing OAE is urgently needed to better understand the potential ecosystem impacts of its use. Because of their inherent complexity, we urge caution when extrapolating our laboratory results to marine ecosystems. Last, to inform societies of the potential benefits and risks of CDR technologies, including OAE, it is imperative to field-test, monitor, and verify their efficacy—methods to accomplish each of these, however, remain in their infancy.

## MATERIALS AND METHODS

### Experimental design

Monospecific cultures of *E. huxleyi* (collected in June 2015 during a coccolithophore bloom within California’s Santa Barbara Basin) and *Chaetoceros* sp. (collected in November 2014 off the coast of Monterey Bay, CA) were grown at 15°C in nutrient-enriched filtered seawater under cool fluorescent light (photon flux density of ~170 μmol m^−2^ s^−1^; 16/8-hour light/dark cycle). Cultures were gently bubbled with 420 (±2%) parts per million by volume CO_2_ balanced air (Airgas) to simulate modern ocean-atmosphere gas exchange. All culture media were prepared in acid-rinsed 20 liters of polycarbonate (PC) carboys using natural seawater collected from the Santa Barbara Basin (Global Positioning System coordinates provided in table S4). The seawater was enriched with 100 μM nitrate, 6.24 μM phosphate, 70 μM silicate, and f/2 concentrations of vitamins and trace metals (*50*, *51*). To simulate calcium-containing alkalinity enhancement, calculated amounts of Na_2_CO_3_ and CaCl_2_H_4_O_2_ (both ACS certified) were added to treatment media. TA was raised to ~3000 μmol kg^−1^ and ~5000 μmol kg^−1^ in the moderate- and high-TA treatments, respectively. Selected values were based on model-predicted OAE scenarios that provide estimated seawater carbonate chemistry conditions following extensive global alkalinity addition combined with emissions reduction and CDR and rapid alkalinity addition—which could result in moderate-to-severe localized effects on ocean chemistry (*52*).

Following nutrient addition and TA enhancement, culture media were filtered through a 0.22-μm sterile polyethersulfone Steritop filter (Millipore) and stored overnight (~12 hours) at room temperature. The following morning, pseudo-triplicate samples were collected from each carboy, and 1-liter aliquots of filtered culture media were distributed into 2-liter Nalgene PC bottles with ventilation caps connected to an air manifold system using silicone tubing. Autoclaved 0.2-μm PC air filters (Omicron) were placed immediately upstream of the ventilation ports to prevent contamination.

To bring the carbonate system conditions within the range of model-predicted OAE scenarios (*52*), the culture media were bubbled before inoculation with phytoplankton cells. The *E. huxleyi* and *Chaetoceros* sp. experiments were bubbled for 3 and 4 days, respectively, before cell inoculation—we adapted the onset of each experiment to the growth stage of our stock cultures to ensure cells were in exponential growth upon inoculation. All 2-liter PC bottles—except for nine negative controls (three for each control and TA treatment level)—were inoculated using stock cultures of *E. huxleyi* (~1500 cells ml^−1^) or *Chaetoceros* sp. (~500 cells ml^−1^). Inoculum cells were not pre-acclimated to treatment conditions.

Sample collection began immediately after cell inoculation (day 0) and was repeated at predetermined time points over the course of each experiment—triplicate independent experimental replicates were sacrificed for each treatment level at each sampling time point. Experimental timeframes were designed to ensure that samples were only collected during the exponential phase of growth under nutrient-replete conditions to avoid the confounding effect of nutrient limitation. We inoculated culture media with exponentially growing phytoplankton cells and continued each experiment until cells had been exposed to experimental conditions for >7 generations, a commonly applied acclimation period during coccolithophore and diatom culturing experiments (*43*, *53*, *54*)—although species such as *E. huxleyi* have been shown to acclimate more quickly (*55*). Sample collection was consistently initiated at 11:00 a.m. (± 30 min) PT to avoid the effects of the photocycle on measured parameters.

### Carbonate chemistry analysis

Carbonate system parameters were obtained via direct measurements of TA, pH, salinity, temperature, and dissolved inorganic nutrients. The remaining carbonate system parameters were calculated with CO2sys (*56*) using refit equilibrium constants from Mehrbach *et al.* (*57*, *58*) and the total pH scale.

Carbonate chemistry samples were filtered through a 0.2-μm PC filter into 250-ml borosilicate bottles via gentle suction with a peristaltic pump following established protocols (*59*). Samples were then preserved with 100 μl of HgCl_2_ and stored at room temperature until the time of analysis. TA was measured with a Mettler Toledo T5 titrator following the open-cell titration protocols outlined by Dickson *et al.* (*60*). Salinity was measured with a YSI 3100 conductivity probe. pH was measured with a Shimadzu UV-1280 spectrophotometer using calibrated m-cresol purple dye (*61*).

### Microscopy and photochemical efficiency

Phytoplankton cell abundances (cells per milliliter) were estimated with an Olympus BX53 light microscope and a 1-ml Sedgewick-Rafter counting chamber. Samples for SEM-EDX were collected on 0.4-μm PC filters, dried at room temperature, and analyzed using a Zeiss EVO 10 LS with EDS detector and its accompanying Smart EDX software at the Santa Barbara Museum of Natural History. The photochemical efficiency of photosystem II—or variable-to-maximum fluorescence (*F*_v_/*F*_m_)—was assessed via OJIP using an AquaPen-C AP 110-C fluorometer after dark-adapting cells for 20 min before analysis.

### POC, PON, nutrients, PIC, and BSi

Samples for POC and PON were collected on precombusted 25-mm glass microfiber filters (GF/F, Whatman) and stored at −20°C until analysis via automated organic element analysis using the dumas combustion method (CEC 440HA, Exeter Analytical). Cellular POC and PON values were normalized using phytoplankton cell abundances. Dissolved inorganic nutrient concentrations (nitrate + nitrite, ammonia, phosphate, and silicic acid) were quantified via flow injection analysis (QuikChem 8000 Series 2, Zellweger Analytics). Both analyses were conducted by the Universiy of California, Santa Barbara, Marine Science Institute Analytical Lab.

During our *E. huxleyi* experiment, cellular PIC (i.e., CaCO_3_) samples were collected and processed following the procedures of Iglesias-Rodriguez *et al.* (*59*). Particulate and dissolved calcium and sodium ion concentrations were analyzed via inductively coupled plasma optical-emission spectrometry using a PerkinElmer Optima 7300DV at the University of California, Riverside, Environmental Sciences Research Laboratory. Residual calcium from seawater contamination on our filters was corrected using dissolved sodium concentrations (*62*). Cellular PIC was normalized using *E. huxleyi* cell abundances.

During our *Chaetoceros sp.* and abiotic experiments, BSi samples were collected on 0.6-μm PC filters and stored frozen until analysis using the NaOH digestion method described by Krause *et al.* (*63*). BSi samples collected during the *Chaetoceros* sp. experiment were corrected for the influence of abiotic mineral precipitates using mean silica values from days 3 to 11 of the abiotic experiment (silica values from day 0 of the abiotic experiment were excluded as media were inoculated with cells following 3 to 4days of bubbling during the two biotic experiments): 1.06 μM (control), 1.37 μM (moderate TA), and 1.07 μM (high TA). BSi values were normalized using *Chaetoceros* sp. cell abundances.

### Statistical analysis

Specific growth rates (μ, days^−1^) and generation times (days) were calculated by fitting natural logarithm transformed cell abundances per unit time to a linear regression model. All other variables were statistically analyzed via Spearman’s correlation (*r*). All statistical analyses were performed in RStudio Build 351—R version 4.1.2 (2021-11-01).

## Notes

View/request a protocol for this paper from *Bio-protocol*.
