# Supplementary material for: Coccolithophores and diatoms resilient to ocean alkalinity enhancement: A glimpse of hope?
Source: Sci Adv. 2023 Jun 14;9(24):eadg6066. doi: 10.1126/sciadv.adg6066 (PMC10266724; doi:10.1126/sciadv.adg6066)
Supplement: Supplementary file 1 — Figs. S1 to S7 Tables S1 to S4 Legends for data S1 and S2 [file sciadv.adg6066_sm.pdf]

Science Advances
AAAS

Supplementary Materials for

Coccolithophores and diatoms resilient to ocean alkalinity enhancement: A glimpse of hope?

James A. Gately *et al.*

Corresponding author: James A. Gately, jgately@ucsb.edu

Sci. Adv. **9**, eadg6066 (2023)
DOI: 10.1126/sciadv.adg6066

The PDF file includes:

Figs. S1 to S7
Tables S1 to S4
Legends for data S1 and S2

Other Supplementary Material for this manuscript includes the following:

Data S1 and S2

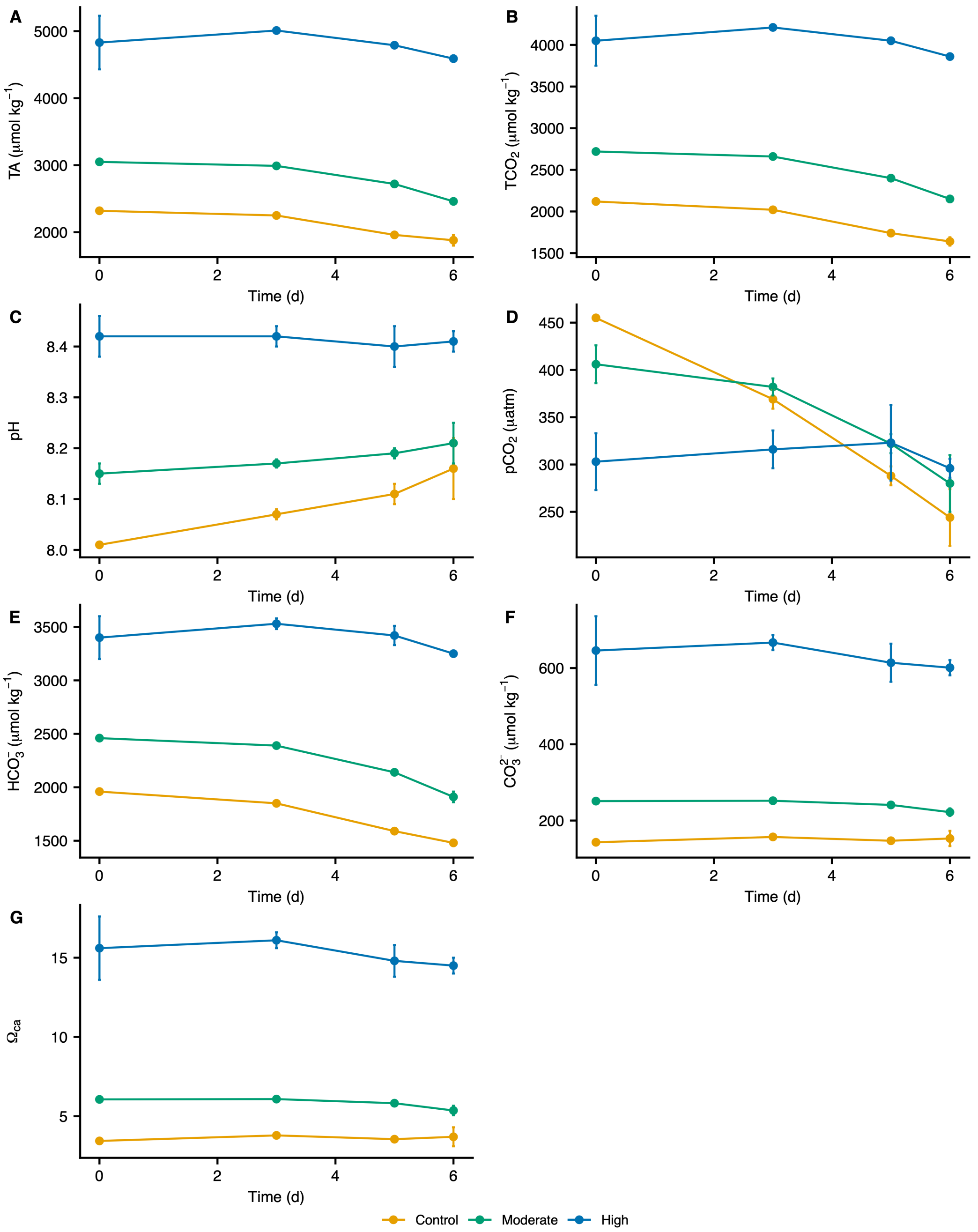

Fig. S1. Evolution of carbonate chemistry parameters during *E. huxleyi* OAE culturing experiment. We show here the evolution of the carbonate system in our control (yellow), moderate-TA (green), and high-TA (blue) cultures. Time 0 is the time at which the culture media was inoculated with *E. huxleyi* cells (i.e., initial TA spike + 3 days of bubbling with air containing 420 (± 2%) ppm pCO_2). Mean values are displayed with error bars representing one standard deviation (n = 3). See Data S2 for values at each sampling timepoint.

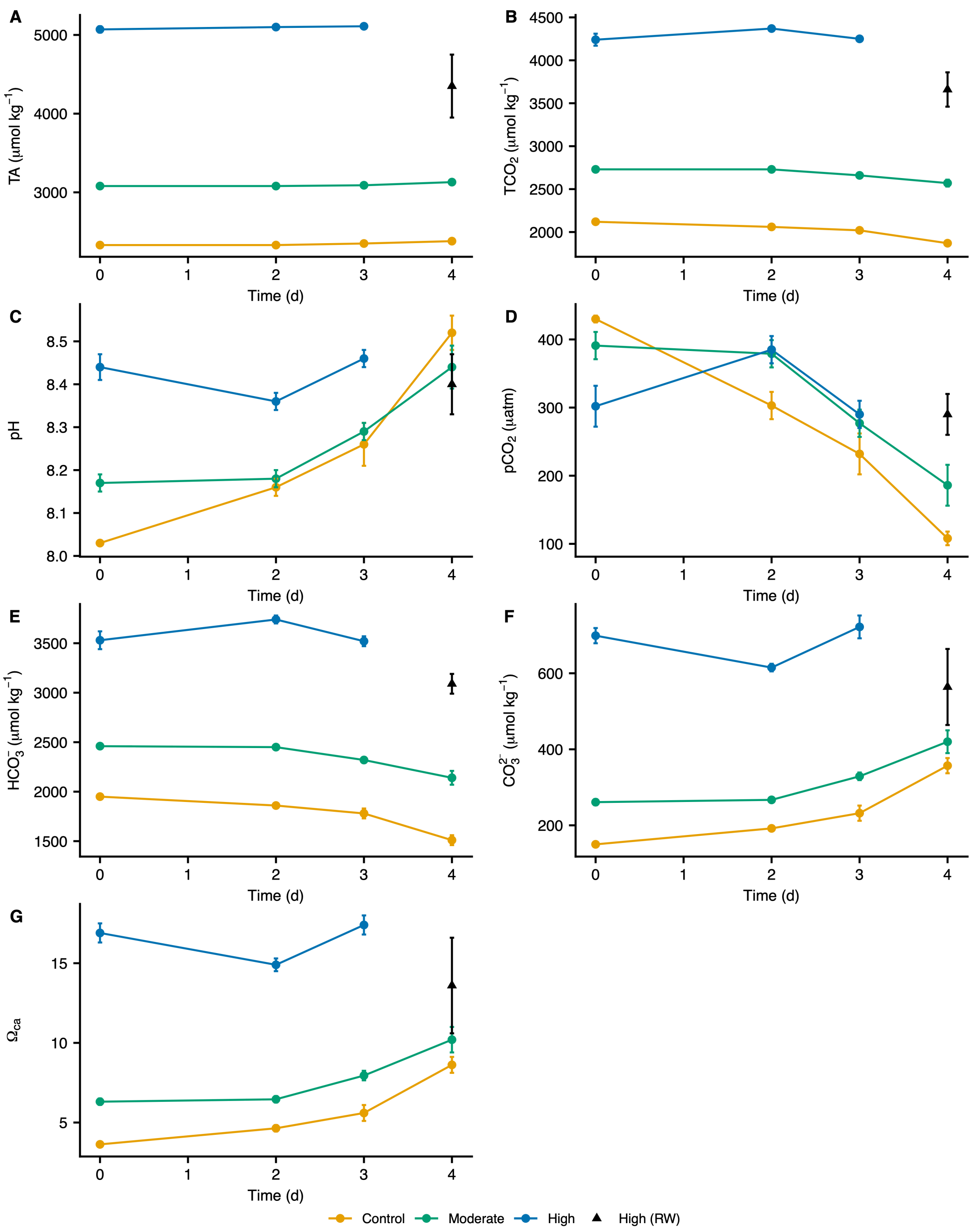

Fig. S2. Evolution of carbonate chemistry parameters during *Chaetoceros* sp. OAE culturing experiment. We show here the evolution of the carbonate system in our control (yellow), moderate-TA (green), and high-TA (blue) cultures. Black triangles indicate high-TA samples that reverse weathered during sample storage. Time 0 is the time at which the culture media was inoculated with *Chaetoceros* sp. cells (i.e., initial TA spike + 4 days of bubbling with air containing 420 (± 2%) ppm pCO_2). Mean values are displayed with error bars representing one standard deviation (n = 3). See Data S2 for values at each sampling timepoint.

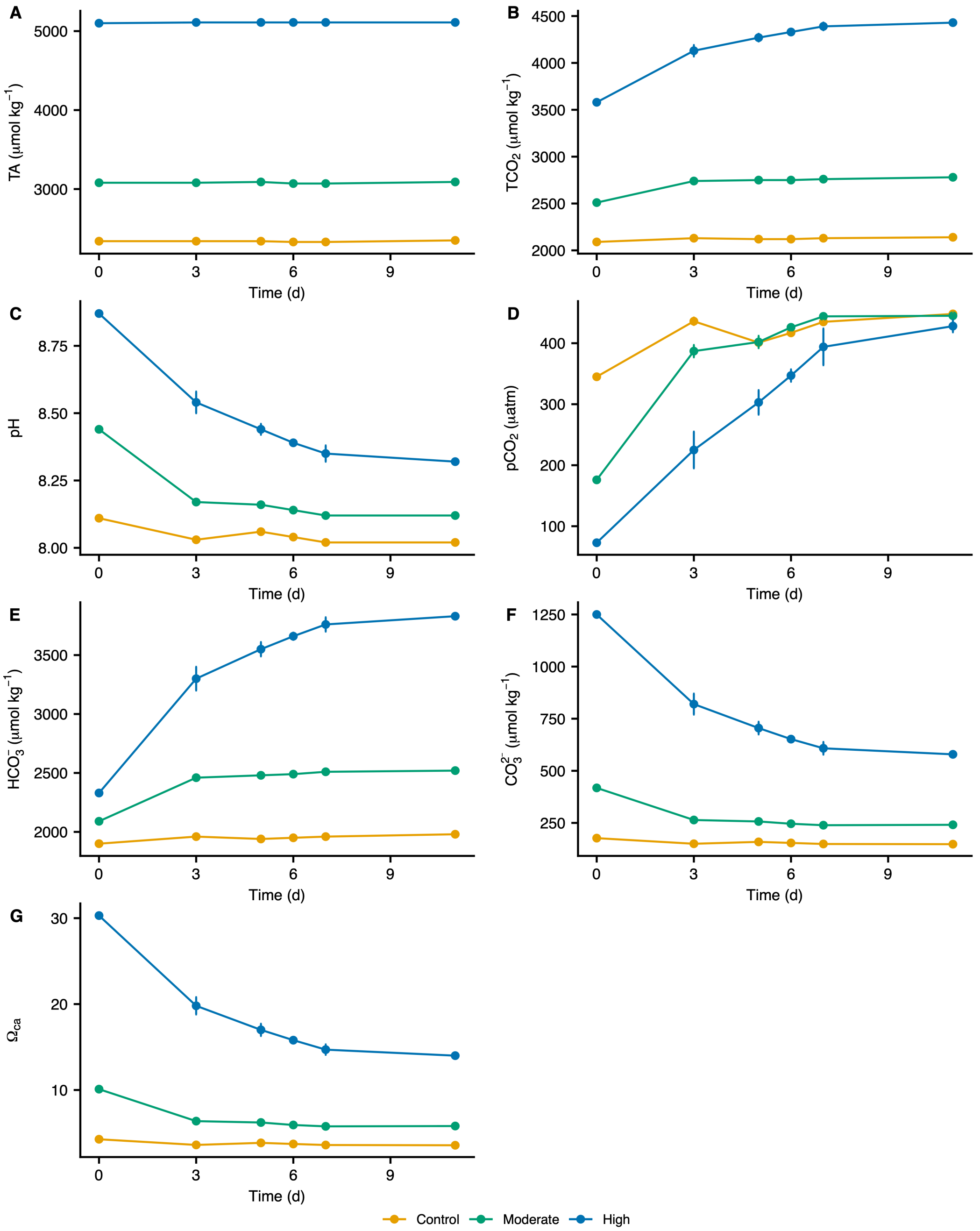

Fig. S3. Evolution of carbonate chemistry parameters during abiotic OAE experiment. We show here the evolution of the carbonate system in the control (yellow), moderate-TA (green), and high-TA (blue) media. Mean values are displayed with error bars representing one standard deviation (n = 3). See Data S1 for values at each sampling timepoint.

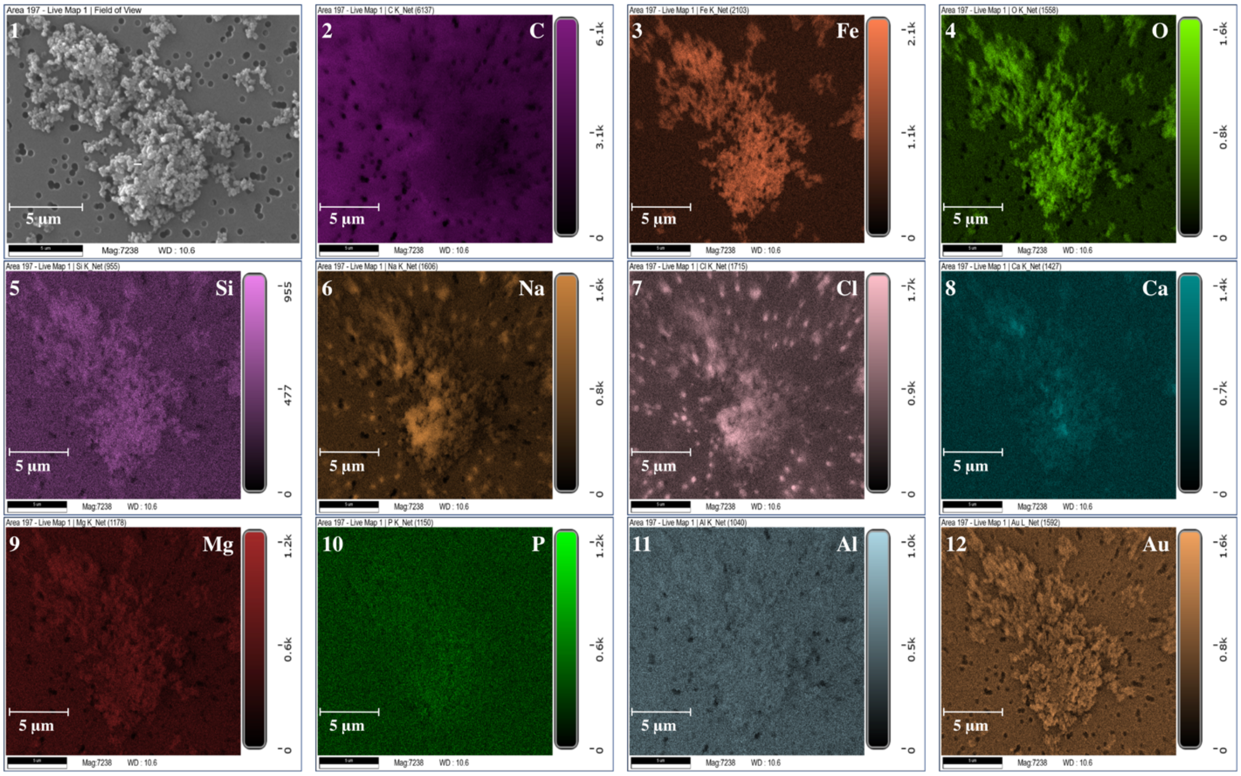

Fig. S4. SEM-EDX elemental mappings of mineral precipitate from high-TA treatment media collected during the abiotic OAE experiment. Image 1 is an SEM image of the mineral precipitate. Images 2-12 are X-ray mappings for each noted element. Background C from the filter can be seen in image 2. Filters were not milli-Q washed – which led to NaCl contamination (6, 7) – and were coated with Au (12) during analysis.

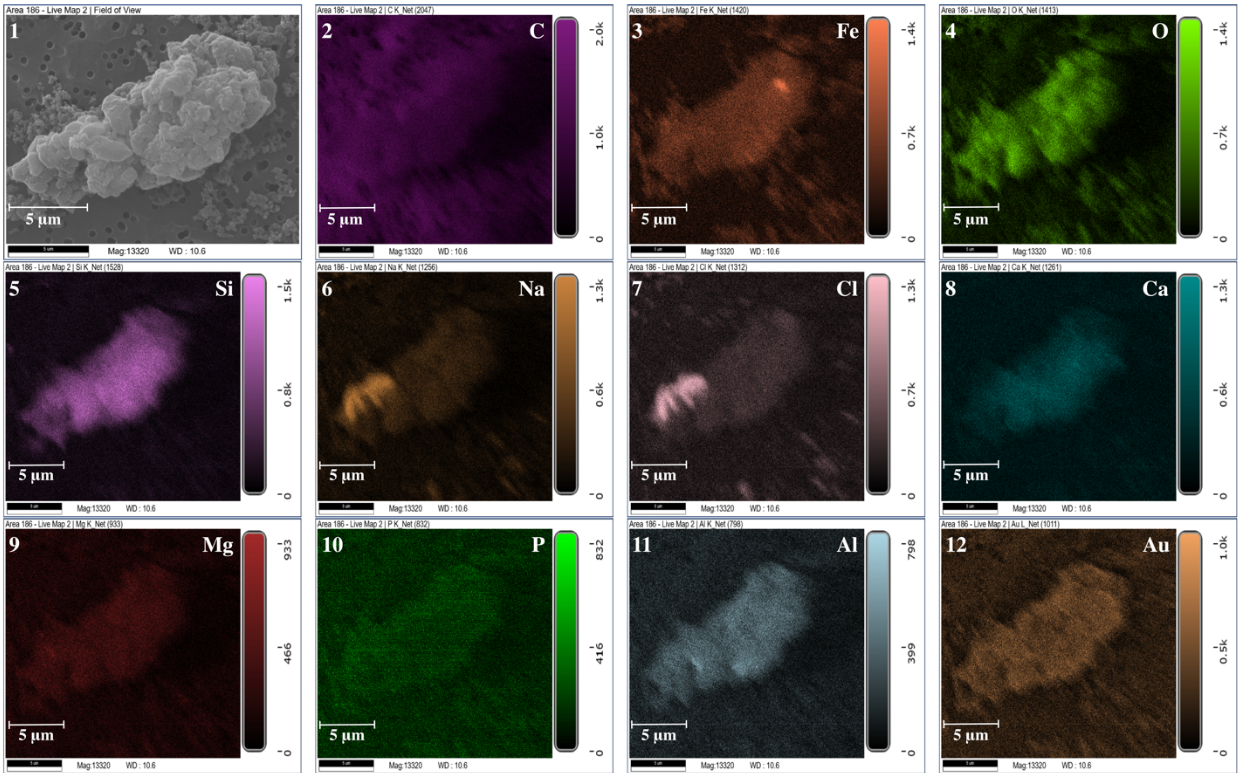

Fig. S5. SEM-EDX elemental mappings of mineral precipitate from high-TA treatment collected during the abiotic OAE experiment. Image 1 is an SEM image of the mineral precipitate. Images 2-12 are X-ray mappings for each noted element. Background C from the filter can be seen in image 2. Filters were not milli-Q washed, which lead to NaCl contamination (6, 7), and coated with Au (12) for analysis.

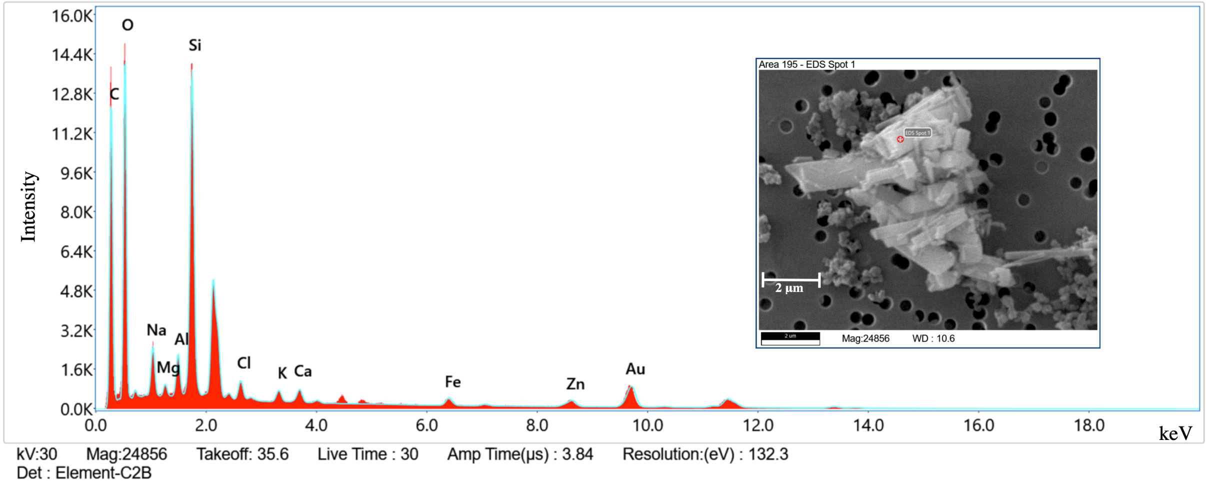

Fig. S6. SEM-EDX spot analysis spectrum of mineral precipitate from high-TA treatment media during the abiotic OAE experiment. EDX spot analysis revealed a mineral precipitate composed primarily of Si and O. Two additional peaks are clearly evident: background C from the filter, and Au due to the filter being coated with gold for analysis. Filters were not milli-Q washed, which resulted in NaCl contamination. Minor elemental peaks are also visible in the spectrum (e.g., Fe, Mg), but their peaks are small relative to those discussed above.

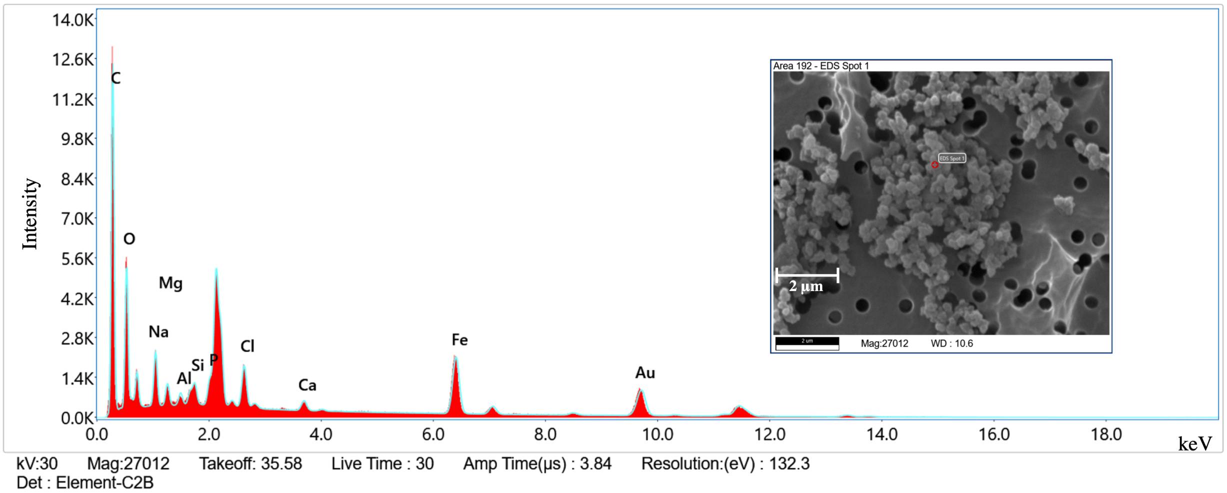

Fig. S7. SEM-EDX spot analysis spectrum of mineral precipitate from control media during the abiotic OAE experiment. EDX spot analysis revealed a mineral precipitate composed primarily of Fe and O. Two additional peaks are clearly evident: background C from the filter, and Au due to the filter being coated with gold for analysis. Filters were not milli-Q washed, which resulted in NaCl contamination. Minor elemental peaks are also visible in the spectrum, but their peaks are small relative to those discussed above.

Table S1.
Dissolved inorganic nutrient concentrations during *Emiliania huxleyi* and *Chaetoceros* sp. OAE culturing experiments.

Experiment	Day	OAE Group	DIN (μmol L^{-1})	DIP (μmol L^{-1})	DSi (μmol L^{-1})
Emiliania huxleyi	0*	Control	101.8 (0.78)	4.69 (0.06)	71.63 (1.03)
		Moderate	103.17 (0.5)	4.69 (0.02)	68.3 (0.2)
		High	104.17 (0.32)	4.9 (0.03)	59.1 (0.17)
	3	Control	93.87 (0.93)	3.53 (0.08)	73 (0.75)
		Moderate	96.03 (1.23)	4.06 (0.06)	68.27 (0.29)
		High	98.87 (0.59)	4.52 (0.03)	59.17 (0.06)
	5	Control	64.87 (1.79)	1.38 (0.12)	73.23 (0.29)
		Moderate	70.77 (1.36)	2.05 (0.13)	68.3 (0.44)
		High	78.4 (1.39)	2.89 (0.08)	59.23 (0.25)
	6	Control	52.73 (7.54)	0.8 (0.31)	72.54 (0.31)
		Moderate	45.63 (1.66)	0.54 (0.04)	67.45 (0.39)
		High	62.83 (4.05)	1.53 (0.26)	58.55 (0.57)
Chaetoceros sp.	0†	Control	101.07 (0.5)	4.49 (0.05)	71.37 (0.35)
		Moderate	100.87 (0.38)	4.55 (0.03)	66.65 (0.12)
		High	100.7 (0.1)	4.85 (0.05)	59.27 (0.26)
	2	Control	95.63 (0.99)	3.43 (0.14)	68.67 (0.57)
		Moderate	95.8 (0.95)	3.85 (0.1)	64.54 (0.43)
		High	96.27 (0.21)	4.33 (0.06)	57.41 (0.29)
	3	Control	80.33 (3.55)	1.64 (0.45)	59.12 (2.17)
		Moderate	83.57 (2.12)	2.53 (0.38)	57.43 (1.48)
		High	84.97 (1.86)	3.01 (0.25)	50.49 (1.53)
	4	Control	44.13 (1.77)	0.08 (0.05)	33.75 (3)
		Moderate	52.03 (5.06)	0.05 (0)	36.97 (3.44)
		High	39.23 (8.95)	0.07 (0.01)	19.77 (6.48)

Notes:
Mean values are given with one standard deviation in parentheses (n = 3)
Method detection limit (MDL): 0.20 μmol L^{-1} (DIN), 0.10 μmol L^{-1} (DIP), 1.0 μmol L^{-1}
* Day of inoculation = initial TA spike +3 days of bubbling with air containing 420 ppm CO_2
† Day of inoculation = initial TA spike +4 days of bubbling with air containing 420 ppm CO_2

Table S2.
DIP and DSi concentrations during abiotic OAE experiments.

Experiment	Day	OAE Group	DIP (µmol L^{-1})	ΔDIP (%)	DSi (µmol L^{-1})	ΔDSi (%)
Abiotic	0	Control	5.99 (0.07)	--	77.37 (0.5)	--
		Moderate	5.32 (0.03)	-11 (1)	72.72 (0.2)	-6 (1)
		High	4.92 (0.04)	-18 (1)	66.96 (0.09)	-13 (1)
	3	Control	4.92 (0.1)	--	77.92 (0.3)	--
		Moderate	4.74 (0.02)	-4 (2)	73.84 (0.3)	-5 (1)
		High	4.93 (0.2)	0 (5)	66.8 (0.2)	-14 (0)
	5	Control	4.27 (0.03)	--	77.25 (0.4)	--
		Moderate	4.7 (0.02)	10 (1)	73.58 (0.2)	-5 (1)
		High	5.06 (0.1)	19 (2)	66.6 (0.05)	-14 (1)
	6	Control	4.33 (0.04)	--	77.3 (0.09)	--
		Moderate	4.64 (0.08)	7 (2)	73.6 (0.09)	-5 (0)
		High	5.03 (0.01)	16 (1)	66.79 (0.3)	-14 (0)
	7	Control	4.2 (0.04)	--	77.12 (0.2)	--
		Moderate	4.66 (0.03)	11 (1)	73.24 (0.2)	-5 (0)
		High	4.96 (0.2)	18 (5)	66.94 (0.05)	-13 (0)
	11	Control	4.14 (0.04)	--	77.41 (0.1)	--
		Moderate	4.61 (0.03)	11 (1)	73.5 (0.2)	-5 (0)
		High	4.75 (0.1)	15 (3)	66.94 (0.2)	-14 (0)

Notes:
Mean values are given with one standard deviation in parentheses (n = 3)
One control sample was lost on Day 0 (n = 2)
Differences are relative to controls
Method detection limit (MDL): 0.10 µmol L-1 (DIP), 1.0 µmol L^{-1} (DSi)

Table S3.
F_v/F_m during *Emiliania huxleyi* and *Chaetoceros* sp. OAE culturing experiments (all time points excluding day 0).

Experiment	OAE Group	F_v/F_m	F_v/F_m (%)
Emiliania huxleyi	Control	0.63 (0.01)	--
	Moderate	0.63 (0.01)	0 (NaN)
	High	0.62 (0.01)	-2 (2)
Chaetoceros sp.	Control	0.67 (0.01)	--
	Moderate	0.66 (0.01)	-1 (2)
	High	0.65 (0.01)	-3 (2)

Notes:
Mean values are given with one standard deviation in parentheses (n = 9)
Differences are relative to controls

Table S4.
Seawater collection date and GPS coordinates.

Experiment	Seawater Collection Date	GPS Coordinates
Emiliania huxleyi	2021-10-13	34 22.549N 119 47.633W
Chaetoceros sp.	2021-11-16	34 20.466N 119 48.578W
Abiotic	2022-03-09	34 18.526N 119 47.612W

Data S1. (separate file)
Evolution of carbonate chemistry parameters during abiotic OAE experiment.

Data S2. (separate file)
Carbonate chemistry parameters for OAE culturing experiments.
